# Reactive Sintering of Dysprosium-Iron Garnet via a Perovskite-Hematite Solid State Reaction and Physical Properties of the Material

**DOI:** 10.3390/ma15072356

**Published:** 2022-03-22

**Authors:** Magdalena Stan, Radosław Lach, Paweł A. Krawczyk, Wojciech Salamon, Jakub Haberko, Jacek Nizioł, Anita Trenczek-Zając, Łukasz Gondek, Błażej Kowalski, Antoni Żywczak

**Affiliations:** 1 Faculty of Materials Science and Ceramics, AGH University of Science and Technology, Al. Mickiewicza 30, 30-059 Krakow, Poland; mstan@agh.edu.pl (M.S.); radoslaw.lach@agh.edu.pl (R.L.); pkrawczy@agh.edu.pl (P.A.K.); anita.trenczek-zajac@agh.edu.pl (A.T.-Z.); 2 Academic Centre for Materials and Nanotechnology, AGH University of Science and Technology, Al. Mickiewicza 30, 30-059 Krakow, Poland; salamon@agh.edu.pl; 3 Faculty of Physics and Applied Computer Science, AGH University of Science and Technology, Al. Mickiewicza 30, 30-059 Krakow, Poland; haberko@fis.agh.edu.pl (J.H.); niziol@fis.agh.edu.pl (J.N.); lgondek@agh.edu.pl (Ł.G.); 4 Faculty of Electrical Engineering, Automatics, Computer Science and Biomedical Engineering, AGH University of Science and Technology, Al. Mickiewicza 30, 30-059 Krakow, Poland; blazej.k97@gmail.com

**Keywords:** rare-earth iron garnet, synthesis, magnetic properties, dielectric properties

## Abstract

In this paper, we report on a successful synthesis of dysprosium iron garnet Dy_3_Fe_5_O_12_ (DyIG) by a reactive synthesis method involving dysprosium iron perovskite and hematite. Phase formation was traced using dilatometry, and XRD measurements attested to the formation of the desired structure. Samples with relative density close to 97% were fabricated. The samples were characterized using vibrating sample magnetometry, dielectric spectroscopy, and UV-Vis-NIR spectroscopy. Magnetic properties were probed in temperatures between 80 and 700 K with a maximum applied field of 1 kOe. The measurements revealed several effects: the compensation of magnetic moments at a certain temperature, the inversion of the magnetocaloric effect, and the ability to measure the Curie temperature of the material. Activation energy was determined from UV-Vis-NIR and dielectric spectroscopy measurements. Characteristic magnetic temperatures and activation energy values of the samples were similar to bulk DyIG obtained using other methods.

## 1. Introduction

Dysprosium-iron-garnet and other rare-earth iron garnets (RE-IG) are materials that exhibit numerous attractive physical properties involving magnetic [1], ferroelectric [2], optical [3], magnetoelectric [4], and magnetodielectric phenomena [5]. They also demonstrate other effects of excellent engineering interest: magnetocaloric [6,7,8], Barnett [9], spin Seebeck [10,11], and Faraday effect [5]. Therefore, they either show strong potential or have already found applications in diverse areas of technology, e.g., memory devices [12]. In the latter publication, magnetization control using electrical current has been shown. Furthermore, RE-IGs with perpendicular magnetic anisotropy could be successfully used in spintronics for polarization control [13]. Other applications include waveguide optical isolators and Faraday rotators [14], magnonic devices [15], microwave devices [16], and sub-GHz wireless applications [17]. Moreover, they can be utilized in magnetic refrigeration devices [18], laser sources of radiation [19], electrochemical hydrogen storage [20], or as microwave components–circulators [21] (in this case, the authors have proven that their rare-earth iron garnet material is suitable for operation in the K_α_ band in the communication industry), antennas, isolators, and so on [22].

Several methods have been developed for synthesizing yttrium iron garnet and its rare-earth containing counterparts. These include co-precipitation [23,24], the sol-gel method [25,26,27], high-energy ball milling [28], mechanochemical processing [29], glycine assisted combustion method [30], citrate and solid-state reaction method [31], anion resin exchange precipitation method [19], or the micro-pulling-down method [32]. The most common way to obtain rare-earth iron garnets is reactive sintering between oxides [33]. Typically, in this method, stoichiometric amounts of oxides are mixed together, finely ground, then annealed (pre-sintered), ground again, pressed into pellets, and, finally, sintered in the target temperature. Thus, this method requires two stages of prolonged grinding, which can adversely affect the purity of the material. Moreover, both the pre-sintering and the final sintering times necessary to obtain dense samples are typically very long, in the range of 12 h. Another popular technique, the sol-gel method [8], suffers from similar disadvantages, which renders these synthesis routes time- and energy-consuming. In this work, we aimed to obtain dense polycrystalline dysprosium-iron-garnet material via a reactive sintering method based on a solid-state reaction [34] between dysprosium-iron perovskite (DyFeO_3_) and iron oxide, α-Fe_2_O_3_. We have verified [35] that a similar approach is effective in synthesizing a gadolinium-iron garnet.

In cases of sintering that involve solid-state reactions, the most advantageous situation takes place when products have a larger molar volume compared to reactants. This leads to the sealing of unfilled pores, greatly increasing bulk density. This phenomenon can be observed in DyFeO_3_ and α-Fe_2_O_3_ reactive sintering. The reaction equation can be written as:

(1) $$3{\mathrm{DyFeO}}_{3}+{\mathrm{Fe}}_{2}O_{3}\to {\mathrm{Dy}}_{3}{\mathrm{Fe}}_{5}O_{12}$$

with the total molar volume of the reactants (135.60 cm^3^/mol) smaller than that of the product (145.04 cm^3^/mol).

In contrast, both in the synthesis route involving reactive sintering between oxides and the sintering of precursors obtained by co-precipitation, this favorable effect of pore filling is not present for the following reason: the first step in these syntheses is the perovskite (DyFeO_3_) formation. However, in this reaction:

(2) $${\mathrm{Dy}}_{2}O_{3}+{\mathrm{Fe}}_{2}O_{3}\to 2{\mathrm{DyFeO}}_{3}$$

The substrates are characterized by larger molar volume (78.23 cm^3^/g) than the perovskite product (70.09 cm^3^/g). This, in turn, is less advantageous from the point of view of pore filling, leads to sample shrinkage, and may even result in macroscopic cracks in the sample. We have observed similar phenomena in the case of gadolinium-iron garnet synthesis (GdIG) [34].

Although contrarily to the sintering of oxides, the proposed method requires an additional step of rare-earth perovskite powder preparation, the successive sintering stage is relatively fast and leads to a high-quality, dense ceramic material. In this work, we show that this methodology is valid also for DyIG, which possibly opens a route for facile synthesis of a broader class of rare-earth iron garnets. Furthermore, we characterize the obtained material in terms of its magnetic and dielectric properties.

## 2. Experimental

The first step was to obtain a powder of dysprosium-iron perovskite precursor. Dysporosium (III) oxide (Qingdao Xiguanya Mining Industry Co., Ltd., Qingdao, China, 99.99%) was first dissolved in 65% HNO_3_ and then mixed with a solution of Fe(NO_3_)_3_ (Chempur, AR, 99.99%), with the solution concentrations reflecting the stoichiometry of DyFeO_3_. Following this, precipitation was carried out in a stirred ammonia solution with pH below 11. The resulting powder was washed multiple times with distilled water in order to remove nitrate ions, dried, and, finally, calcined at 1200 °C for 2 h. Hematite (α-Fe_2_O_3_) powder was obtained separately by precipitation from iron (III) nitrate (Chempur, AR) in ammonia solution, followed by a heat treatment at 300 °C for 2 h. These two powders were then mixed at proportions corresponding to the stoichiometry of the final garnet and homogenized in isopropyl alcohol for 24 h in a roller mixer. The specific surface areas of the powders were probed via the BET adsorption method using a NOVA 1200e apparatus (Quantachrome Instruments) with nitrogen at T = 77 K as adsorbate. Average grain size was then calculated based on these measurements and the density of the material (DyFeO_3_—7.60 g/cm^3^, Fe_2_O_3_—5.24 g/cm^3^). The obtained powder was pressed into one-axis cylindrical pellets with a diameter of 13 mm and a height of 3 mm at a pressure of 50 MPa. The pellets were isostatically repressed at a pressure of 250 MPa. The compacts were then sintered in an air atmosphere at 1200, 1300, and 1400 °C, with a rate of temperature increase of 10 °C/min and 2 h soaking time.

X-ray powder diffraction patterns were collected using a MalvernPanalytical Empyrean diffractometer operating with Cu X-ray tube (K_α_ radiation). The instrumental line profiles were calibrated using a NIST 600 standard sample. The data were analyzed by the Rietveld method implemented in the FullProf Suite package [36].

The powder densification process via compact shrinkage was followed using a dilatometer (DIL 402C, NETZSCH). Hydrostatic weighing allowed us to determine the apparent density of the sintered samples. SEM images were taken using a Scanning Electron Microscope (FEI Nova Nano SEM 200). The polished specimens were then thermally etched at 1200 °C with 2 h soaking time. The average grain size of the sintered garnet was calculated according to the method described by Mendelson et al. [37].

Magnetization as a function of temperature from 80 to 300 K was measured using a LakeShore Model 7407 vibrating sample magnetometer equipped with a cryostat in liquid nitrogen. Measurements within the temperature range from 300 K to 1250 K were performed using the oven in an argon atmosphere (99.9995% purity) with a heating rate of 5 °C∙min^−1^ in 0.1 T magnetic field.

UV-Vis-NIR measurements were carried out using a JASCO 670 spectrophotometer, equipped with a 150 mm integrating sphere.

Broadband dielectric spectroscopy (BDS) measurements were carried out using an MFIA 5 MHz impedance analyzer from Zurich Instruments and a custom-made cryostat. The sample was measured in the plane capacitor geometry. The upper electrode was 10 mm in diameter, i.e., less than the sample, to minimize stray field effects at the capacitor edge. Initially, the sample was cooled down to −50 °C, then equilibrated in temperatures increasing in 10 °C intervals up to T = 200 °C. At each temperature increment, dielectric parameters of the sample were measured (in terms of absolute impedance and loss tangent) to the applied alternating field (300 mV_RMS_) at frequencies ranging from 1 Hz to 1 MHz in 10 logarithmic steps per decade.

## 3. Results and Discussion

### 3.1. Synthesis

The specific surface area of the precursor powders, as well as the particle sizes, are presented in Table 1.

The large disparity between particle sizes apparent from the above measurements is beneficial for the material’s densification, as smaller iron oxide particles may fill the gaps between the larger DyIP ones. Moreover, smaller particles will have a tendency to accumulate on the surface of larger ones, increasing the number of contacts between the reactants and promoting diffusion. All these factors should facilitate the solid-state reaction.

**Figure 1:**
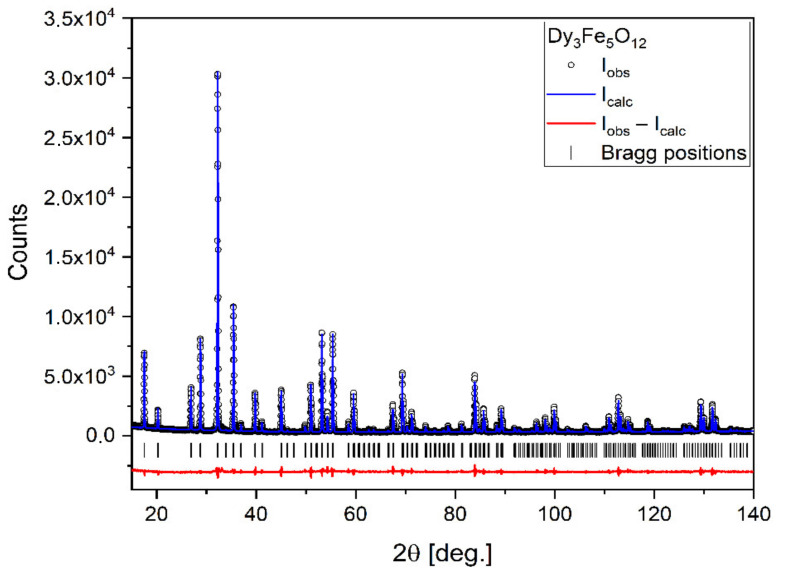
X-ray diffraction pattern of Dy_3_Fe_5_O_12_ with results of the Rietveld refinement.

X-ray diffraction (XRD) analysis was performed on a powder sample obtained from heating the mixture of powders up to 1100 °C. This allowed us to confirm that the solid-state reaction between the perovskite and the iron oxide indeed took place to yield the desired garnet structure (Figure 1). Another XRD analysis performed on a sample sintered at 1400 °C yielded the same result (XRD pattern not included here), which attests to the fact that no further chemical transformation of the material takes place between 1100 °C and 1400 °C.

The XRD pattern reveals that the synthesized sample is of very high purity. All observed reflections can be described by the cubic $Ia\bar{3}d$ space group (No. 230). The lattice parameter was found to be a = 12.40645(7) Å. The atoms occupy the following positions:

Dy: the 24c position with fractional coordinates 1/8; 0; 1/4;
Fe1: the 16a position with fractional coordinates 0; 0; 0;
Fe2: the 24d position with fractional coordinates 3/8; 0; 1/4;
O: the 96 h position with fractional coordinates 0.3497(2); −0.0305(2) −0.0555(2).

The crystallite size was extracted from XRD peak widths using the Williamson–Hall method and was equal to 360 ± 30 nm.

**Figure 2:**
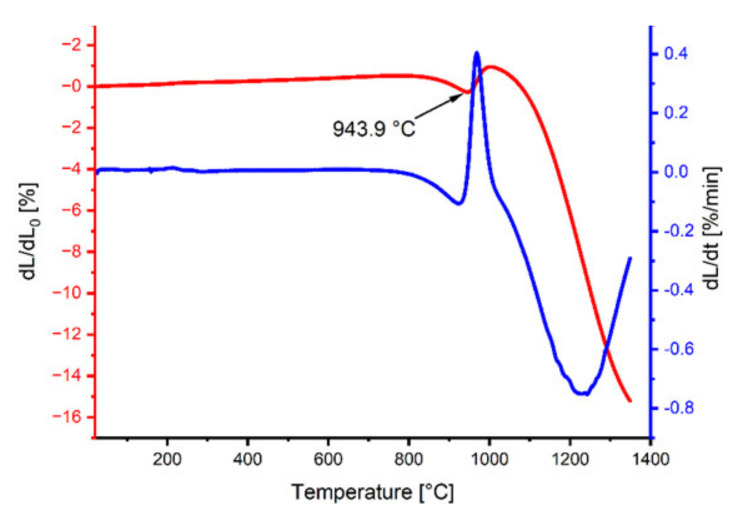
Dilatometric and derivative curves of the DyFeO_3_–Fe_2_O_3_ mixture.

The analysis of the dilatometric curves (Figure 2) allowed the assessment of the sample’s shrinkage as a function of temperature. At a temperature of 943.9 °C, we observed a linear decrease in the sample size, which corresponds to the reaction of the dysprosium iron garnet formation. The rate of temperature rise in the case of powder heating for the X-ray diffraction analysis and shrinkage measurement of the test materials was the same as for sintering (10 °C/min).

Table 2 presents the relative density of samples sintered at 1200 °C, 1300 °C, and 1400 °C. It is worth noting that a density close to 97% of the theoretical density was achieved at 1400 °C.

**Figure 3:**
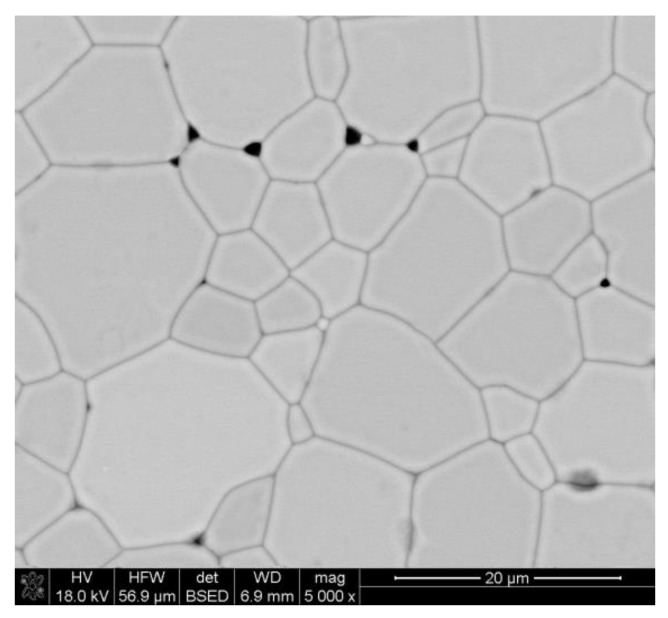
SEM micrographs of the sample sintered at 1400 °C.

Relative density was calculated assuming DyIG (Dy_3_Fe_5_O_12_) density of 6.61 g/cm^3^ [38]. The average grain size was determined from SEM according to methods described in [37], and for the sample sintered at 1400 °C it was equal to 8.91 ± 0.68 µm. A representative SEM micrograph of the garnet sample is presented in Figure 3.

### 3.2. Magnetic Properties

**Figure 4:**
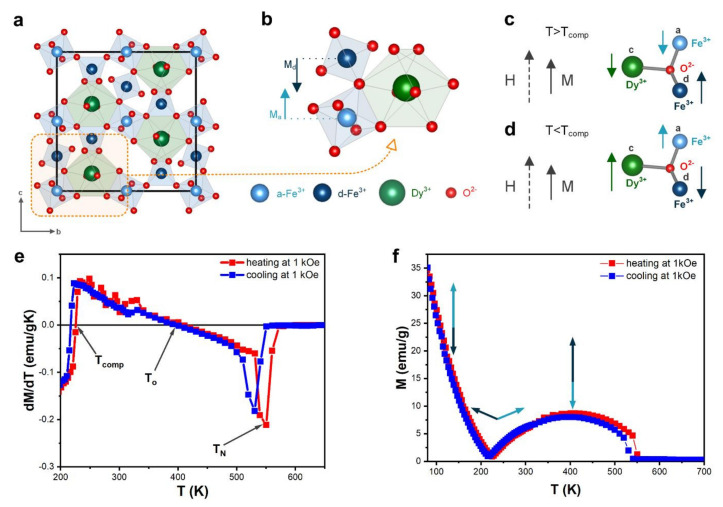
(**a**) The crystal structure of DyIG; (**b**) Unit cell of DyIG; (**c**) Magnetic moments below compensation temperature; (**d**) Magnetic moments above compensation temperature; (**e**) Temperature dependent magnetization at 0.1 T for DyIG; (**f**) Derivative of the temperature-dependence magnetization at 1 kOe was used for determination of characteristic temperatures.

In dysprosium-iron-garnet (DyIG), the metal ions are located in the oxygen polyhedron gap, where Dy ions occupy 24c dodecahedral site, and Fe ions occupy 16a octahedral and 24d tetrahedral sites (Figure 4a). The magnetic moment of Fe comes from the super-exchange interaction between magnetic moments from a-site Fe^3+^ ions and d-site Fe^3+^, which induces antiparallelism between these sites (Figure 4b):

(3) $$M_{\mathrm{Fe}}=\left|M_{\mathrm{Fe},d}-M_{\mathrm{Fe},a}\right|$$

The magnetic moment of dysprosium (M_Dy_) introduced at the c-site is antiparallel to the M_Fe_. The total magnetic moment:

(4) $$M=\left|M_{\mathrm{Fe},d}-M_{\mathrm{Fe},a}\right|-M_{\mathrm{Dy}}$$

DyIG possesses three different magnetic temperatures [39] (Table 3): compensation (T_comp_), inversion magnetocaloric effect (T_o_), and the Curie temperature (T_C_). Their values were estimated from the derivative of the temperature-magnetization dependence.

The exchange field between the Fe and Dy sublattices is much weaker than that between the a-site Fe^3+^ and d-site Fe^3+^ sublattices. At temperatures lower than T_comp_ (Figure 4c), the total magnetization of DyIG is determined by the Dy sublattice magnetic moment located opposite the Fe sublattice magnetic moment. As the temperature increases, the absolute values of M_Fe,d_–M_Fe,a_ and F_Dy_ change, and at some point, at T = T_comp_, cancel out to yield M = 0. Table 3 shows our me3asured T_comp_ values in the heating (220 K) and cooling mode (225 K). Several articles reported the values of T_comp_ for DyIG garnet; T_comp_ was found to be equal 215 K [40], 217 K [41], 218 K [9], 218.5 K [5,42], 223 K [43], 261 K [26]. As can be seen from this comparison, the T_comp_ values of our sample are aligned with the majority of reported values.

At temperatures higher than T_comp_ (Figure 4d), the total sample magnetization should be determined by the iron sublattice. The dysprosium sublattice can be essentially regarded as a system of paramagnetic ions situated in an exchange field created by the Fe sublattices, because of the weak interaction between the dysprosium ions.

The garnets, which exhibit compensation temperature, are expected to possess the inverse magnetocaloric effect, because the magnetic order increases with temperature increasing between T_comp_ and T_o_. At the inversion of magnetocaloric effect temperature, magnetization is maximized. T_o_ is equal to 409 K in the heating mode and 396 K in the cooling mode. A similar value close to 400 K was estimated by Nguyet et al. [40], but theoretical calculations of P. J. von Ranke [41] yield a slightly different value (352 K).

The Curie temperature is 531 K and 550 K in the two measurement modes, which is close to literature reports, citing the values of 550 K [40] and 570 K [41].

### 3.3. Activation Energy from UV-VIS

**Figure 5:**
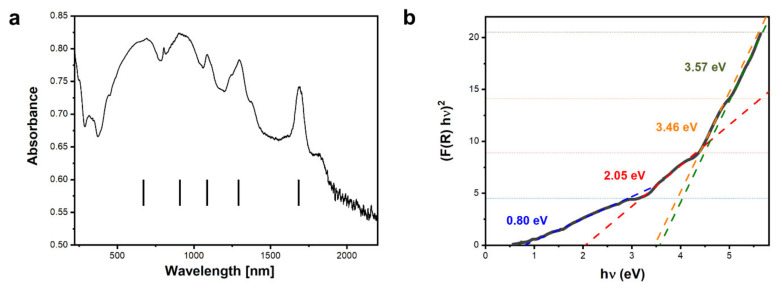
(**a**) UV-Vis-NIR spectroscopy measurement of the DyIG sample and (**b**) fits to the linear regions of the ${\left[F\left(R_{\infty }\right)h\nu \right]}^{2}$ function, linear fits are traced with different colors for better clarity. The colors of E_g_ values provided on the graph correspond to the colors of corresponding fitted lines.

UV-Vis-NIR spectroscopy measurements of the sample (Figure 5a) show a series of maxima. Broad absorption maxima at λ ≈ 670 nm and λ ≈ 910 nm are a result of the presence of Dy^3+^ ions. The sharp peaks at λ ≈ 1086 nm, λ ≈ 1291 nm, and λ ≈ 1685 nm are due to transitions of 4f electrons in Dy and were observed in very similar positions in rare-earth doped yttrium iron garnets [44]. Based on the UV-Vis-NIR results, the activation energy was determined using the Kubelka–Munk approach. The Kubelka–Munk function was calculated:

(5) $$F\left(R_{\infty }\right)=\frac{{\left(1-R_{\infty }\right)}^{2}}{2R_{\infty }}$$

where $R_{\infty }=R_{\mathrm{sample}}/R_{\mathrm{std}}$ and $R_{\mathrm{sample}}$, $R_{\mathrm{std}}$ are the total integrated reflectances of the sample and standard, respectively. The data were then fitted to the equation:

(6) $${\left[F\left(R_{\infty }\right)h\nu \right]}^{2}=C_{2}\left(h\nu -E_{g}\right)$$

and E_g_ values were extracted from the fits. Four distinct linear regions are visible in Figure 5b, which give rise to the following E_g_ values: 0.80 eV, 2.05 eV, 3.46 eV, and 3.57 eV.

### 3.4. Activation Energy from Dielectric Spectroscopy Measurements

**Figure 6:**
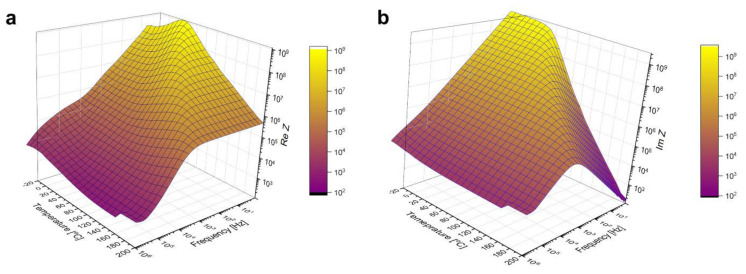
Real (**a**) and imaginary (**b**) part of the sample’s impedance as a function of temperature and frequency, measured by BDS.

**Figure 7:**
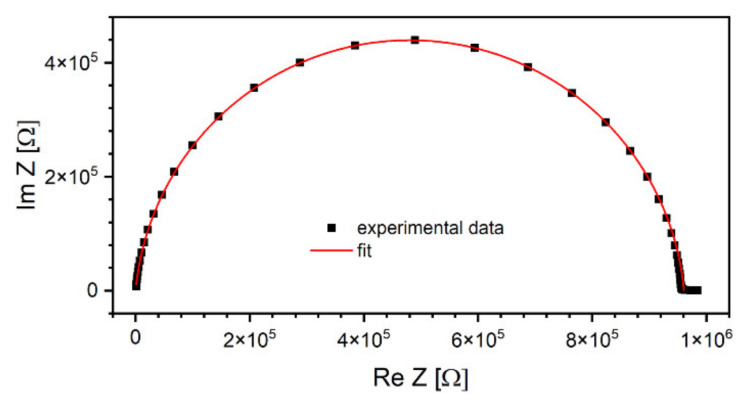
An example of a Cole–Cole plot using the data recorded at T = 180 °C. Right-hand side of the semicircle corresponds to lower frequencies, while the left-hand side to high frequencies [46].

Broadband dielectric spectroscopy measurement results are plotted in Figure 6. If these surfaces were cut along a fixed temperature value, the resulting graphs would be characteristic of a capacitance connected in parallel to a resistance. Taking into account such an assumption, Cole–Cole plots were prepared and fitted using the Equation (7) used to describe depressed arcs that often appear when impedance data on solids is plotted in the $Z^{*}$ plane [45]:

(7) $$Z^{*}\left(\omega \right)=R_{\infty }+\frac{R_{S}-R_{\infty }}{1+{\left(j\omega \tau \right)}^{1-\alpha }}$$

where 0≤α≤1 is an empirical number, $R_{S}$ is static resistance (i.e., extrapolated to ω=0) and $R_{\infty }$ high-frequency resistance. An example of such plot recorded at T = 180 °C and a corresponding fit is shown in Figure 7.

**Figure 8:**
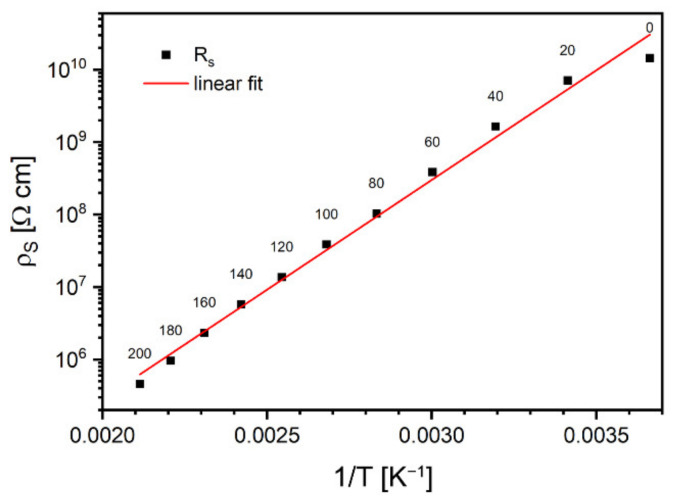
Arrhenius plot of static resistivity. Labels are temperatures in °C.

The advantage of $R_{S}$ deduced from Cole–Cole plots over directly measured with DC techniques resides in reduced influence of parasitic phenomena related to lead contacts, such as electrode polarization. The charge accumulated at interfaces modifies the bias voltage, and, therefore, results of DC measurements become incorrect, especially in the case of materials characterized by non-ohmic conductivity. In contrast, this phenomenon can be neglected with low-frequency alternating fields. Calculated values of the α exponent were centered around 0.05, regardless of the temperature. Based on the obtained $R_{S}$, a plot of the dependence of the specific resistivity of the sample on the inverted temperature was sketched in Figure 8. Experimental data measured below 0 °C were omitted as being too close to the capacity of the impedance meter.

As one can conclude from Figure 8, the observed conductivity represents thermally activated model, which can be described by the Equation (8):

(8) $$\rho \left(T\right)=\rho_{0}\exp\left(\frac{E_{A}}{\mathrm{kT}}\right)$$

where activation energy, $E_{A}$ was calculated as 0.60 ± 0.02 eV. 

## 4. Conclusions

This article presents a new method of obtaining dense, polycrystalline, single-phase DyIG material. The analysis of the dilatometric curves combined with XRD analysis allowed us to identify the dysprosium iron garnet formation reaction and to confirm high purity of the resulting material. In particular, XRD analysis has shown that the temperature of 1100 °C is sufficient for the reaction to take place. However, in order to obtain high-density samples, higher temperatures are needed as they promote pore filling. Thus, the resulting powders were reactively sintered at temperatures of 1200 °C, 1300 °C, and 1400 °C. In the material sintered at 1400 °C, density around 97% of the theoretical density was achieved.

The magnetic moment of ferrimagnetic DyIG is obtained by coupling Fe and Dy through indirect super-exchange interaction. Characteristic magnetic temperatures of the DyIG material have been determined, specifically the magnetic compensation temperature, inversion of the magnetocaloric effect temperature and Curie temperature. The values are similar to DyIG bulk synthesized using other chemical methods, which indirectly points to the high-quality of our samples. Optical spectroscopy shows a series of lines typical of Dy^3+^ ions. The low-energy part of the absorption spectrum is in line with the hopping mechanism of electronic transport. The activation energy values were established using several methods: optical absorption and dielectric spectroscopy. The reactive synthesis route presented in this paper leads to high-purity, dense DyIG bulk material with numerous potential applications, such as microwave components. The proposed method, as opposed to other synthesis routes, does not require prolonged grinding nor extremely long sintering times. Taking into account interesting magnetic properties of the material, it could also find application in memory devices. To this end, fabrication of thin DyIG films would be necessary. One possibility would be to use bulk DyIG slabs synthesized using our method as targets in the Pulsed Laser Deposition technique. These experiments are currently underway.

The authors declare that they have no known competing financial interests or personal relationships that could have appeared to influence the work reported in this paper.

## Figures and Tables

**Table 1:** Specific surface area and particle sizes of the reactants.

Materials	S_w_ [m^2^/g]	D_BET_ [nm]
Fe_2_O_3_	86.62	13.7
DyIP (DyFeO_3_)	10.27	76.9

**Table 2:** Relative density [% theoretical] of the sintered samples.

Temperature [°C]	Relative Density [%]
1200	85.22 ± 0.02
1300	93.79 ± 0.05
1400	96.92 ± 0.02

± confidence interval at confidence level 95%.

**Table 3:** Characteristic magnetic temperatures of DyIG.

Process	T_comp_ (K)	T_O_ (K)	T_C_ (K)
Heating	225	409	550
Cooling	220	396	531

## Data Availability

Data is available from the authors upon reasonable request.
